# Social Media Impact of Articles Published by Dermatology Residents During Medical School: Cross-sectional Study

**DOI:** 10.2196/39201

**Published:** 2022-09-12

**Authors:** Austin Huang, Harrison Zhu, Kelvin Zhou, R Parker Kirby, Nina Dasari, Gianmarco A Calderara, Kathryn Cordova, Ryan Sorensen, Anshul Bhatnagar, Soo Jung Kim

**Affiliations:** 1 Department of Dermatology Baylor College of Medicine Houston, TX United States

**Keywords:** Altmetric score, bibliometrics, social media, dermatology, resident, medical student, publication, citation, Altmetric, research quality, publish, impact factor, Scientometrics

## Abstract

**Background:**

The Altmetric score (AS) is a novel measure of publication impact that is calculated by the number of mentions across various social media websites. This method may have advantages over traditional bibliometrics in the context of research by medical students.

**Objective:**

This study aimed to determine whether dermatology matriculants who graduated from higher-ranked medical schools published more articles with greater impact (ie, a higher AS) than those from lower-ranked institutions.

**Methods:**

A PubMed search for articles published by dermatology residents who started medical school in 2020 was conducted. Demographic information and Altmetric data were collected, and medical schools were sorted according to US News’ top-25 and non–top-25 categories.

**Results:**

Residents who completed their medical training at a top-25 institution published more papers (mean 4.93, SD 4.18 vs mean 3.11, SD 3.32; *P*<.001) and accrued a significantly higher total AS (mean 67.9, SD 160 vs mean 22.9, SD 75.9; *P*<.001) and average AS (mean 13.1, SD 23.7 vs mean 6.71, SD 32.3; *P*<.001) per article than those who graduated from non–top-25 schools.

**Conclusions:**

Our results indicate that students in top-25 schools may have greater access to research resources and opportunities. With a pass/fail United States Medical Licensing Examination Step 1 exam that may increasingly shift focus toward scholarly output from medical students, further discussion on how to create a more equitable dermatology match is essential.

## Introduction

The Altmetric score (AS) is a novel measure of publication impact that is calculated through an automated algorithm using the number of mentions on numerous social media websites, including Twitter and Facebook [1,2]. It may be advantageous to traditional bibliometrics in the context of analyzing research by medical students, as the AS peaks relatively quickly and measures qualitative data [3,4]. Currently, the relationship between medical school rank and the quality of articles published by dermatology matriculants is unknown.

## Methods

A PubMed search for articles published by dermatology residents who began medical school in 2020 was conducted. Residents who graduated from an osteopathic or foreign medical school, as well as those with a PhD, and articles without a DOI (digital object identifier) were excluded. Demographic information was obtained from publicly available profiles, and AS data were collected from the Altmetric website [1]. Medical schools were sorted into US News’ top-25 and non–top-25 categories, which were ranked partially based on the amount of federal funding received [5,6]. Kruskal-Wallis tests were used to analyze the association between medical school rank and research productivity.

## Results

Postgraduate year 3 dermatology residents (N=401) published 1400 articles during medical school, averaging 3.69 per resident. The mean total AS of articles by each resident was 37.2, with each of their articles averaging an AS of 8.72 (Table 1). Residents who completed their medical training at a top-25 institution published more articles (mean 4.93 vs 3.11, *P*<.001) and accrued a significantly higher total AS (mean 67.9 vs 22.9, *P*<.001) and average AS (mean 13.1 vs 6.71, *P*<.001) per article than those who graduated from non–top-25 schools.

**Table 1:** Altmetric data for research by dermatology residents in medical school.

		Residents, n (%)	Publications, mean (SD)	Total AS^a^, mean (SD)	AS, mean (SD)
All residents		401 (100)	3.69 (3.7)	37.2 (111)	8.72 (30)
**Medical school**					
	Top 25	127 (31.7)	4.93 (4.2)	67.9 (160)	13.1 (23.7)
	Non–top 25	274 (68.3)	3.11 (3.3)	22.9 (75.9)	6.71 (32.3)

^a^AS: Altmetric score.

## Discussion

To our knowledge, this is the first study to use the AS to analyze the impact of articles published by dermatology residents during medical school. Prior groups have assessed the correlation between the AS and citation count [3,4]. Others have used traditional bibliometrics to evaluate medical student research productivity in various fields [7,8]. Since there is only a short time period when students can publish before applying for residency, metrics that take years to accumulate, such as citation count and the h-index, are not reliable for assessment of publication impact during medical school [3]. Given the rise of virtual information sharing due to the COVID-19 pandemic, alternative measurements of publication impact that rely on social media dissemination may become more pertinent. 

Limitations to this study include the inability to capture all articles published due to name changes for various reasons including marriage and divorce, which may disproportionately affect the perceived productivity of female residents [9].

The new pass/fail United States Medical Licensing Examination Step 1 exam, while intended to cultivate the prospect of a holistic application process, has prompted concerns of an increasingly unhealthy focus on medical student scholarly output in research-heavy fields such as dermatology [10]. Our results indicate that students in top-25 schools may have greater access to research resources and opportunities. Students at non–top-25 institutions who cannot afford to take research years may not have a fair opportunity to compete with students from top-25 schools. While not a new phenomenon, the consequences of this new system appear to be antagonistic against the current movement toward equity in the field of medicine. Further discussion on how to create a more equitable match is essential. In a time when dissemination of research through the internet is growing at a rapid pace, we encourage future work to explore the utility of the AS in dermatology.

## Abbreviations

AS: Altmetric score
DOI: digital object identifier
